# Genome-Wide Analysis of *FAR-RELATED SEQUENCES* (*FRS*) Genes Related to Light Response in Soybean (*Glycine max*)

**DOI:** 10.3390/ijms27062638

**Published:** 2026-03-13

**Authors:** Sujun Ye, Lixin Ma, Yinhua Lv, Wenmin Lin, Yang Tang, Xiaoya Lin

**Affiliations:** Guangdong Key Laboratory of Plant Adaptation and Molecular Design, Guangzhou Key Laboratory of Crop Gene Editing, Innovative Center of Molecular Genetics and Evolution, School of Life Sciences, Guangzhou University, Guangzhou 510006, China

**Keywords:** *Glycine max*, *FRS* gene family, genome-wide analysis, light response, shade avoidance response

## Abstract

The *FAR1-RELATED SEQUENCES (FRS)* gene family plays a crucial role in light signaling, stress adaptation, and developmental regulation processes directly impacting crop growth and yield. This study identified 49 *GmFRS* genes unevenly distributed across 17 soybean chromosomes, phylogenetically classified into seven subgroups (I–VII), with subgroup VII forming an exclusive evolutionary subgroup alongside orthologs from Poaceae and Solanaceae. Members within each subfamily share conserved motif compositions and similar exon/intron structures. Gene duplication and selection pressure analyses revealed that the *GmFRS* family expanded primarily through WGD duplication events and then non-syntenic gene duplication, with all members evolving under purifying selection. Promoter analysis identified abundant *cis*-acting elements implicated in responses to light, phytohormones and other abiotic stimuli. Organ-/tissue-specific expression profiling demonstrated organ-preferential expression for family members, with the highest transcript levels observed in flowers (32.7%). Quantitative real-time PCR (qRT-PCR) analysis further indicated that the expression of most *GmFRS* genes is light-inducible and exhibits marked sensitivity to far-red light. This study may elucidate soybean *FRS* family functions in light signaling, development, and stress adaptation, while also providing foundational insights for molecular breeding in *Glycine max*.

## 1. Introduction

Light fuels photosynthesis and conveys environmental cues [1,2,3]. In *Arabidopsis thaliana* (L.) Heynh., the far-red photoreceptor phytochrome A (phyA) toggles between the inactive form (Pr) and active form (Pfr) [4]. Upon far-red light illumination, phyA is translocated to the nucleus by FAR-RED ELONGATED HYPOCOTYL1 (FHY1) and FHY1-LIKE (FHL) transporters. This nuclear accumulation process is a key step in phyA signal transduction and ultimately achieves a physiological response by regulating downstream gene expression [5,6,7]. This nuclear import process is controlled by the transposase-derived plant-specific transcription factors FAR-RED ELONGATED HYPOCOTYL 3 (FHY3) and FAR-RED-IMPAIRED RESPONSE 1 (FAR1), founding members of the highly conserved *FAR1-RELATED SEQUENCES* (*FRS*) family, which directly activate *FHY1/FHL* transcription [8,9,10,11]. Through this molecular mechanism, FHY3 and FAR1 not only participate in plant light signaling but also play an important role in developmental processes such as flowering regulation, branching formation, and shade avoidance responses, which constitute the core regulatory network of plant response to far-red light signals [8,9,10,11].

The FRS family is a class of plant-specific transcription factors derived from mutator-like element (MULE) transposases. Most FRS family members contains three core structural domains: an N-terminal FAR1 DNA-binding domain (containing a zinc finger motif) that specifically recognizes Transposon Terminal Inverted Repeat (TIR) motifs; a centrally located, conserved mutator-like element (MULE) transposase core domain; and a C-terminal SWIM motif (named after SWI2/SNF and MuDR transposases) conferring the transcriptional activation function [8,9,10,11,12]. In *A. thaliana*, 18 members of the AtFRS family have been identified, including twelve FRS and four FRS-RELATED FACTOR (FRF) proteins [8,9,10]. Phylogenetic analyses classify these proteins into six evolutionary subgroups: subgroup I (*AtFHY3*, *AtFAR1*, *AtFRS1*, *AtFRS2*, and *AtFRS4*), subgroup II (*AtFRS6*, *AtFRS8*), subgroup III (*AtFRS7*, *AtFRS12*), subgroup IV (*AtFRS3*, *AtFRS5*, and *AtFRS9*), subgroup V (*AtFRS10*, *AtFRS11*), and subgroup VI (*AtFRF1*, *AtFRF2*, *AtFRF3*, and *AtFRF4*) [8,9,10,11,12,13]. Although these proteins retain conserved sequence homology with the maize MULE transposase (encoded by *MURA* of the MuDR transposon), their genomic loci lack canonical transposon structural features such as TIRs, indicating that the *AtFRS* family has lost transposable activity during plant evolution [14,15]. Notably, AtFRS9 lacks the FAR1 DNA-binding domain but retains the MULE core transposase domain; AtFRS7 and AtFRS12 possess duplicated DNA-binding domains; and AtFRF1, AtFRF2, AtFRF3, and AtFRF4 are defined as structurally truncated members of the FRS family, as they solely retain the FAR1 DNA-binding domain [10]. This structural divergence may be closely related to their functional specialization during evolution [8].

Within the *FRS* family, *FHY3* and *FAR1* have been widely studied in *A. thaliana* and act as a central hub that fuses light perception, stress responses, circadian timing and developmental programs into one streamlined network [13]. They trigger photomorphogenesis by ferrying phyA into the nucleus [16], delay flowering under shade via the miR156–SQUAMOSA-PROMOTER BINDING PROTEIN-LIKE (SPL) module [17], boost thermo and drought tolerance through antioxidant genes [18], lock light input to the circadian clock via the CIRCADIAN CLOCK-ASSOCIATED 1 (CCA1), fine-tune branching by antagonizing SPL-BRC1 [19,20,21], and safeguard chloroplast integrity and starch synthesis [22]. Beyond *FHY3/FAR1*, other *A. thaliana FRS* members (e.g., *AtFRS6* and *AtFRS8*) modulate flowering time and other processes by regulating downstream target transcription [8]. Collectively, they translate environmental cues into growth decisions, underpinning plant phenotypic plasticity.

Soybean (*Glycine max* (L.) Merr.), an important crop, is highly sensitive to photoperiod and light changes [23,24], which influence the flowering time, development, and ultimately yield. Shrinking farmland and a worsening climate expose soybeans to heat and saline–alkaline stresses that erode yield and quality. The *FRS* family, owing to its critical roles in plant growth and development, has been extensively investigated in diverse higher plants, including eucalyptus [18], tea plants [25], walnuts [26], grapes [27], and potatoes [28], with its functions in light signaling, hormone response, and abiotic stress tolerance well-characterized. However, research on the *FRS* family in soybean remains limited. To better understand the *GmFRS* genes, we conducted a genome-wide analysis of the *FRS* gene family in *G. max*, investigating their phylogenetic relationships and genomic features, as well as the physicochemical properties of their encoded proteins. We also analyzed the expression patterns of *FRS* genes under different red to far-red light ratios (R:FR) to predict their functions in response to environmental changes. This study addresses the knowledge gap regarding the *FRS* family in soybean, advances our understanding of its roles in plant physiological processes, and provides a theoretical foundation for soybean breeding and yield improvement.

## 2. Results

### 2.1. Identification and Physicochemical Analysis of GmFRS Gene Family Members

To accurately identify all FRS proteins in *G. max*, we performed BLAST searches against the *G. max* (Williams 82) genome using all experimentally characterized *A. thaliana* FRS protein sequences as queries [8,10]. Initial screening of the *G. max Wm82.a2.v2* genome in Phytozome v13 identified 72 candidate *FRS* genes [29]. However, 25 candidates were unsupported in the updated *G. max Wm82.a2.v4* and *G. max Wm82.a2.v6* assembly and showed no detectable expression in the nine tissues examined [29], suggesting that they likely represent pseudogenes potentially arising from initial assembly errors or misannotation artifacts. To ensure the accuracy of the dataset, all candidate proteins were further verified by HMMER tools to contain at least one of the three conserved domains, SWIM zinc fingers (Pfam: PF04434), FAR1 DNA-binding domain (Pfam: PF03101), or MULE transposase domain (Pfam: PF10551) via InterPro, thereby enhancing identification accuracy. We ultimately identified 49 high-confidence proteins, defining them as the soybean FRS family members (Table S1).

To characterize the structural properties and functional implications of the GmFRS protein family in soybean, we analyzed the GmFRS family proteins and found that they have amino acid numbers ranging from 106 to 880 and molecular weights (Mw) ranging from 11.9 to 99.4 kDa (Table S2). The theoretical isoelectric points (pI) of these proteins ranged from 4.75 to 9.24, including five acidic, 32 neutral, and 12 basic proteins. Notably, 72% of members exhibited pI values between 5.8 and 7.5 (Table S2), suggesting a potential adaptation to cytosolic neutral pH conditions. The grand average of hydropathicity (GRAVY) ranged from −0.855 to −0.306, confirming global hydrophilic characteristics (Table S2). Subcellular localization prediction indicated that the GmFRS proteins were predominantly localized to the nucleus, with the exception of GmFRS45, which was predicted to reside in the plasma membrane (Table S2).

**Figure 1:**
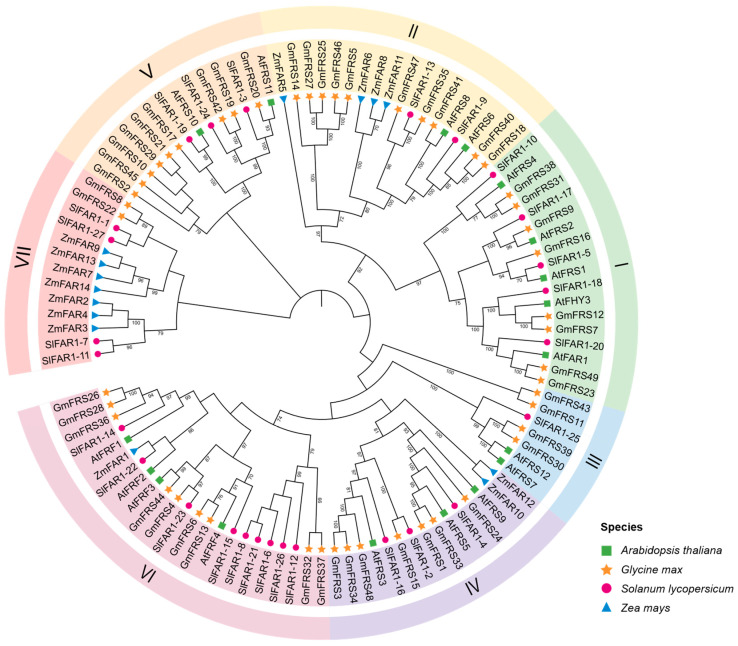
Phylogenetic tree of *GmFRS* gene family members between *Glycine max*, *Z. mays*, *S. lycopersicum*, and *A. thaliana*. Orange stars, blue triangles, red circles, and green squares represent FRS family proteins from *Glycine max*, *Z. mays*, *S. lycopersicum*, and *A. thaliana*, respectively. Different subgroups of proteins are distinguished using different backgrounds. The Roman numerals I–VII denote the subgroups classified based on the phylogenetic analysis of the *A. thaliana FRS* family.

To elucidate the evolutionary relationships of the soybean *FRS* family, we performed multiple sequence alignments of 18 AtFRSs from *A. thaliana* [8,10], 27 SlFRSs from *Solanum lycopersicum* L. [30], 14 ZmFRSs from *Zea mays* L. [31], and 49 GmFRSs from *G. max*. Arabidopsis served as a reference model with well-annotated functions and established phylogenetic relationships, while tomato and maize represented typical eudicot and monocot species, respectively, both supported by high-quality genomic data. By comparing these species spanning key evolutionary nodes, we assessed the evolutionary trajectory and potential functional divergence of the soybean FRS family. Based on subfamily distribution within the *A. thaliana FRS* family, the 108 *FRS* genes were classified into seven subgroups (I-VII) (Figure 1). *GmFRS* members exhibit non-uniform distribution: subgroup I (*n* = 8), subgroup II (*n* = 10), subgroup III (*n* = 4), subgroup IV (*n* = 7), subgroup V (*n* = 9), subgroup VI (*n* = 9), and subgroup VII (*n* = 2). Notably, subgroup VII lacks *A. thaliana* homologs but contains two soybean members (*GmFRS8*, *GmFRS22*) that cluster with *S. lycopersicum* and *Z. mays* proteins under high bootstrap support (>89%). This provides an evolutionary framework for inferring the potential functional conservation and mechanisms of action in GmFRS proteins. Further analysis of functional association revealed the flowering time regulators AtFRS6/AtFRS8, the factors governing light, hormone, and senescence-associated pathways AtFHY3/AtFAR1, and the factor inhibiting hypocotyl elongation under red light have orthologs in soybean (e.g., subgroup I/II members) [8]. Specifically, phylogenetic analysis indicated that AtFHY3 shares the closest relationship with GmFRS7 and GmFRS12, while AtFAR1 is most closely related to GmFRS23 and GmFRS49, suggesting potential functional similarity and positioning these as key members of the GmFRS family. Interspecific evolutionary distance analysis revealed significantly greater divergence between *Z. mays* FRS proteins and soybean homologs compared to other species, consistent with the strong sequence conservation characteristic of the gramineous gene family’s evolution. These analyses of phylogenetic and functional modulars establish an evolutionary framework for deciphering legume-specific functional innovations in FRS proteins and their roles in crop adaptive evolution.

### 2.2. Analysis of Chromosomal Distribution and Duplication of the GmFRS Family Genes

**Figure 2:**
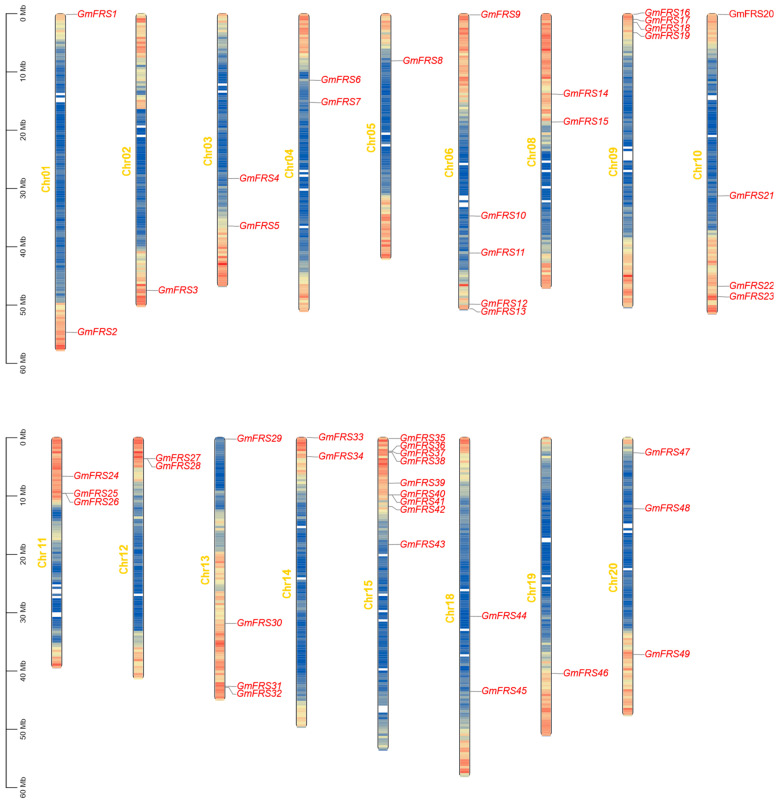
Chromosomal location of *GmFRS* gene family members. The scale on the left of each chromosome indicates physical distance in megabases (Mb). The color gradient along each chromosome (red to blue) represents the distribution density of all annotated genes in the corresponding region, with red indicating high gene-density areas, blue indicating low gene-density areas, and white sections denoting gene-sparse regions. The specific loci of individual *GmFRS* genes are labeled in red.

The chromosomal distribution of *GmFRS* genes was analyzed using the soybean reference genome (*Glycine max Wm82.a4.v1*) with TBtools v2.322 software [32]. A total of 49 *GmFRS* genes were mapped to *G.max* chromosomes and distributed across 17 chromosomes (Figure 2). No genes were detected on Chr09, Chr16, or Chr17. Chr15 harbored the greatest number of *GmFRS* genes, with nine members (18.4% of the total), while Chr06, Chr09–11, and Chr13 contained a moderate number (3–5 genes). The remaining chromosomes (Chr01–05, Chr08, Chr12, Chr14, and Chr18–20) each carried only 1–2 genes. Further spatial distribution analysis revealed the significant enrichment of this gene family in subtelomeric regions, with only a minor fraction localized to mid-chromosomal arm regions (Figure 2).

**Figure 3:**
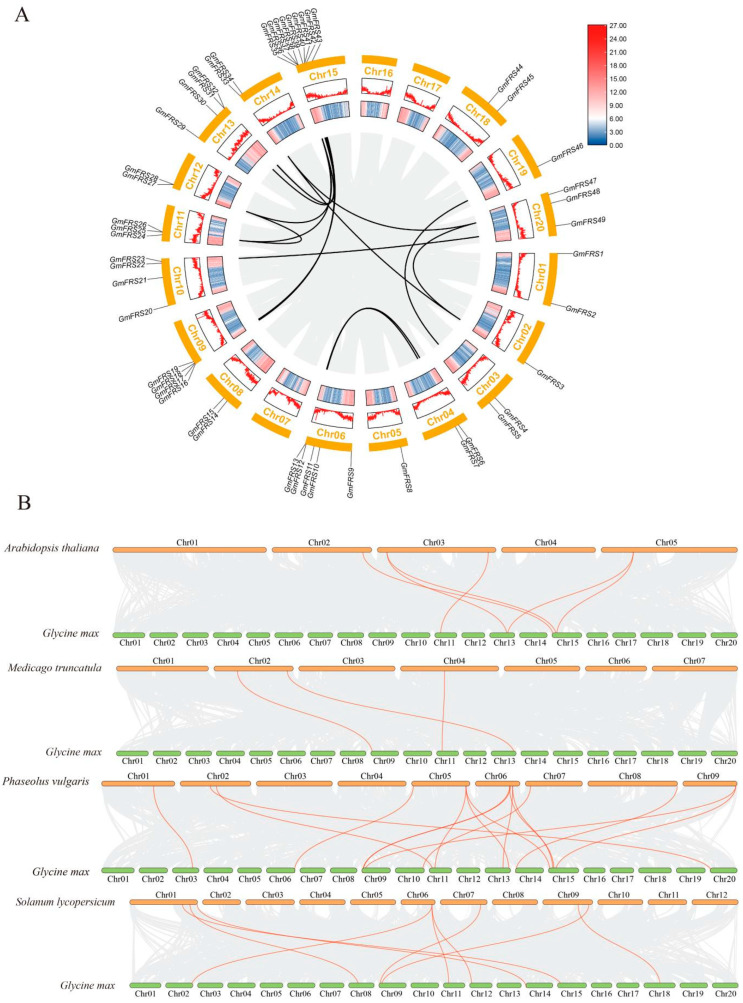
(**A**) Collinearity relationship of *GmFRS* gene family members. The gray lines in the background depict all homologous gene pairs identified by BLASTP (E-value < 1 × 10^−10^, Num of BlastHits = 5), while the highlighted lines (in black) indicate internal collinear gene pairs of the *GmFRS* gene family. (**B**) Synteny analysis of *GmFRS*s between soybeans and four other dicots, including *A. thaliana*, *Medicago sativa*, *Phaseolus vulgaris*, and *S. lycopersicum*. The highlighted lines (in orange) indicate collinear gene pairs between soybeans and the other species.

Tandem and segmental duplications serve as central mechanisms underlying the expansion of gene families [33]. Tandem duplication is characterized by the presence of two closely linked genes on a single chromosome, typically spaced within an interval of five or fewer genes [34]. In contrast, segmental duplication entails the replication of extensive chromosomal regions across the genome, a process frequently linked to chromosomal rearrangements and polyploidization [35]. A total of 15 syntenic gene pairs were identified. Among these, *GmFRS26*, *GmFRS28*, and *GmFRS36* exhibited synteny between each pair, while *GmFRS3*, *GmFRS34*, and *GmFRS48* shared an analogous syntenic relationship (Figure 3). All other genes were present in individual orthologous pairs (Figure 3). This conserved duplication pattern suggests that these genes may hold potential significance in the functional evolution of the family. Collectively, all collinear pairs were identified as segmental/WGD duplications, indicating that these events primarily drove the *GmFRS* family’s expansion. The expansion of the gene family may have contributed to the functional specialization observed in the *GmFRS* genes, potentially playing a role in the soybean’s adaptive evolution.

To elucidate the evolutionary history of the *FRS* gene family, we conducted a collinearity analysis between soybeans and related eudicots (*A. thaliana*, *Phaseolus vulgaris* L., *S. lycopersicum*, and *Medicago truncatula Gaertn*) and monocots (*Sorghum bicolor L., Moench*, *Oryza sativa* L., *Z. mays*, and *Saccharum spontaneum*). No syntenic orthologs of *GmFRS* genes were detected in the monocots (Figure S1). In contrast, syntenic paralogs were found in all four eudicots, with six, 16, six, and three collinear gene pairs identified in *A. thaliana*, *P. vulgaris*, *S. lycopersicum*, and *M. truncatula*, respectively (Figure 3B; Table S9). Notably, *GmFRS26* was syntenic in all four eudicots, indicating high evolutionary conservation and a potential core function (Table S9). These results imply that the *FRS* gene family originated after the monocot–eudicot divergence.

The Ka/Ks ratio is a metric used to quantify selective pressure in molecular evolution. A ratio less than 1 indicates purifying selection, which removes deleterious mutations to conserve protein function and stability; a ratio greater than 1 signifies positive selection, where natural selection drives the rapid fixation of beneficial mutations, thereby accelerating gene evolution; whereas a ratio equal to 1 suggests neutral evolution, indicating that mutations are fixed randomly without the influence of natural selection [36]. In this study, Ka/Ks ratios for all gene pairs were significantly less than 1 (0.116 < Ks < 0.403) (Table S4). These evolutionarily constrained patterns align with characteristics of functionally essential genes, suggesting that the *GmFRS* family likely performs core biological functions important for adaptive evolution.

### 2.3. Analysis of Conserved Domains and Gene Structure in the GmFRS Family

**Figure 4:**
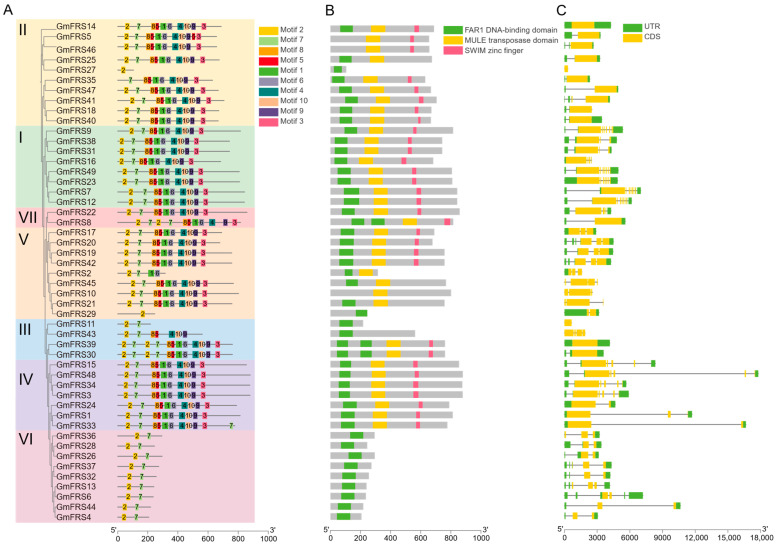
Gene structure, motif analysis, and conserved domains of GmFRSs. (**A**) The evolutionary relationship and conserved motifs of GmFRS proteins. The Roman numerals I-VII denote the subgroups classified based on the phylogenetic analysis of the *A. thaliana FRS* family. At the bottom, the scale bars denote the lengths of the protein sequences. (**B**) Distribution of essential domains of *GmFRS* family members. The scale bars denote the lengths of the protein sequences. Different colored boxes represent conserved domains, while gray regions indicate non-conserved regions. (**C**) The gene structures of *GmFRS* genes are illustrated, with yellow boxes representing the coding sequences (CDS), black lines indicating introns, and green boxes depicting UTR. The scale bars at the bottom indicate the lengths of the genomic sequences.

To further investigate structural characteristics of the *GmFRS* family, we analyzed gene structures, conserved motifs, and domains across all 49 sequences using MEME (Figure 4), identifying 10 conserved motifs in GmFRS proteins (Figure 4A). Conserved motif analysis revealed that most GmFRS proteins share identical motif types and arrangements. In *Arabidopsis*, the four members of subfamily VI (AtFRF1-4) only retain the FAR1 DNA-binding domain. The GmFRS proteins homologous to *Arabidopsis* subfamily VI contain only motif 2 and motif 7. It is hypothesized that these two motifs constitute the conserved FAR1 DNA-binding domain. Furthermore, based on the distribution of conserved domains and the absence of certain domains in some proteins (e.g., GmFRS2 and GmFRS43), we speculate that motif 1 and motif 6 may constitute the MULE transposase domain, while motif 3 constitutes the SWIM zinc finger domain. Additionally, within subfamily III, some GmFRS members possess two copies each of motif 2 and motif 7, correspondingly containing two copies of the FAR1 DNA-binding domain, which is consistent with the structure observed in *Arabidopsis* subfamily III. Domain architecture analysis revealed six distinct types of protein domain organization within the GmFRS family (Figure 4B). Approximately 59.18% of the proteins retained the three characteristic domains, predominantly represented in subgroup I and subgroup IV. GmFRS8, GmFRS39, and GmFRS30 possess two DNA-binding domains. In contrast, GmFRS5 and GmFRS46 lack the FAR1 DNA-binding domain entirely. A third type, including GmFRS25, GmFRS45, and GmFRS21, lacks the SWIM zinc finger domain. GmFRS10 is unique for retaining only the MULE transposase domain. Finally, the members of subfamily VI are truncated proteins solely containing the FAR1 DNA-binding domain. This diversification in domain composition indicates potential functional differentiation among the GmFRS subfamilies.

Analysis of gene structures revealed that *GmFRS* genes within the same subgroup share a relatively conserved exon–intron organization (Figure 4C), which shows a significant correlation with the composition of conserved protein domains. Members in subgroups I, II, and IV, which contain three conserved domains, possess one to seven exons, with most characterized by a notably long exon. It is noteworthy that, within subgroup V, the four members *GmFRS17*, *GmFRS20*, *GmFRS19*, and *GmFRS42* share a similar gene structure consisting of three exons. This suggests the possibility that the three conserved domains may be encoded by three distinct exons. In contrast, members of subgroup VI, which retain only the FAR1 DNA-binding domain, exhibit a markedly simplified gene structure characterized by fewer and shorter exons alongside elongated introns. This genomic architecture aligns with evolution through the loss of entire domain-encoding modules. Furthermore, variations in untranslated regions (UTRs) were observed. *GmFRS36* lacks a 5′ UTR, *GmFRS18* and *GmFRS2* lack a 3′ UTR, and seven members (*GmFRS10*, *GmFRS*11, *GmFRS*21, *GmFRS*27, *GmFRS*29, *GmFRS*43, and *GmFRS*45) lack both, which may influence their transcriptional regulation. Collectively, the dynamic gain and loss of exons/introns, coupled with the modular encoding of conserved protein domains, constitute a flexible genomic architecture. This architecture likely underlies the development of complex regulatory functions, enabling plants to respond to environmental signals [37,38].

### 2.4. Structural Features of GmFRS Proteins

Secondary structures of 49 GmFRS proteins in soybean were predicted using online prediction tools. The results revealed that the major secondary structures of GmFRS proteins include α-helices, β-turns, extended strands, and random coils (Table S3). Among these, α-helices (40.3 ± 6.1%) and random coils (41.6 ± 11.1%) were the predominant components, while extended strands and β-turns accounted for smaller proportions. Notably, GmFRS35 contained 325 amino acids forming α-helices, representing 51.6% of its sequence, whereas GmFRS29 exhibited the highest proportion of random coils (56.1%).

**Figure 5:**
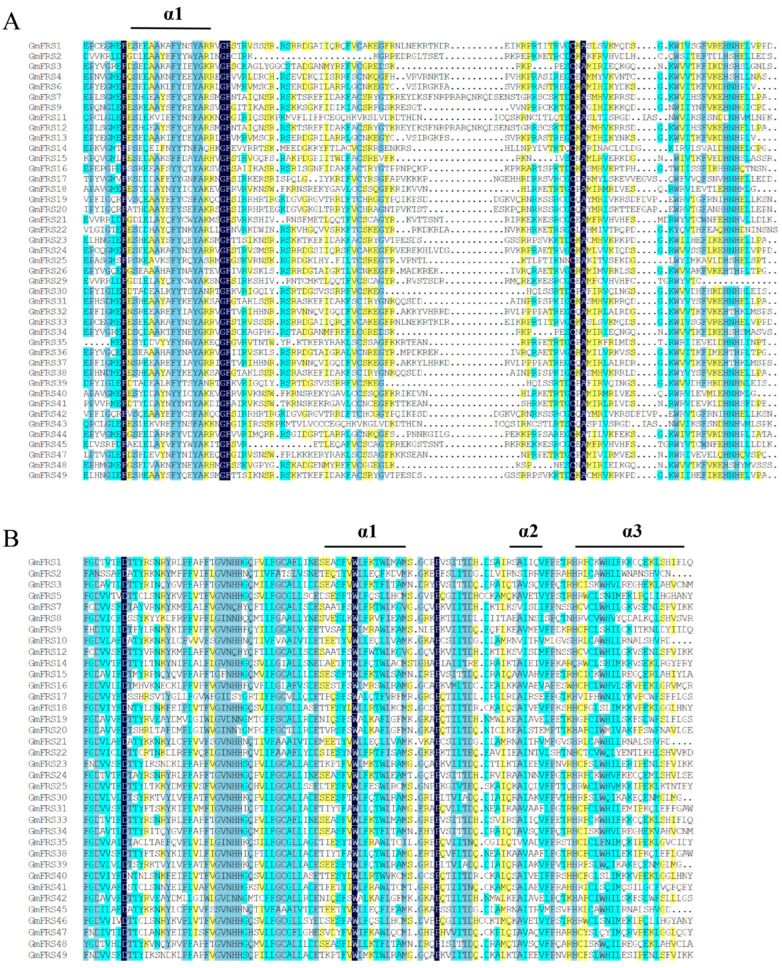
Multiple sequence alignment of FAR1 DNA-binding domain and MULE transposase domain of GmFRSs. (**A**) FAR1 DNA-binding domain. (**B**) MULE transposase domain. The position of the alpha helix is labeled with “α1–α3” in the figure. The color gradient in the alignment indicates sequence conservation, with darker shades representing higher conservation.

To further explore the relationship between conserved domains and secondary structures, multiple sequence alignment of the GmFRS family was performed. The FAR1 DNA-binding domain (approximately 90 amino acids) was localized at the N-terminus of GmFRS proteins and is characterized by an N-terminal α-helix (α1) (Figure 5A). The central MULE transposase domain (~90 amino acids) comprises three α-helices (α1–α3) (Figure 5B). Notably, the conserved domain was absent in GmFRS2, GmFRS29, and GmFRS11, with only the FAR1 DNA-binding domain retained; these proteins exhibited a significantly lower proportion of α-helices compared to other members within the same subgroup.

**Figure 6:**
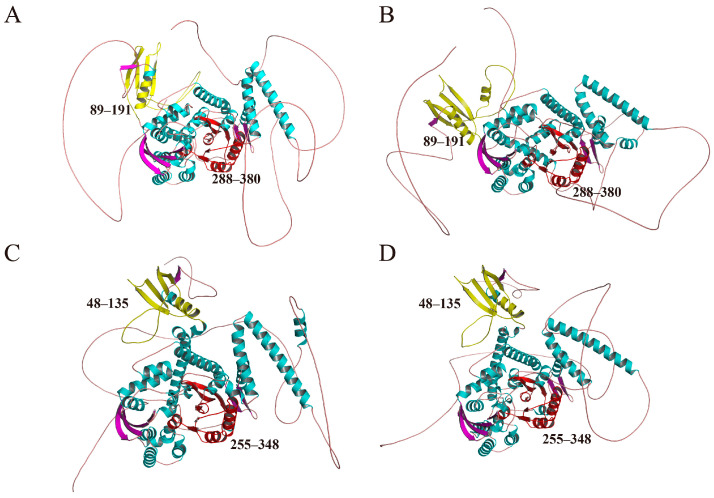
Predicted tertiary structures of GmFRS7, GmFRS12, GmFRS23, and GmFRS49. (**A**) Predicted three-dimensional structure of the GmFRS7 protein. (**B**) Predicted three-dimensional structure of the GmFRS12 protein. (**C**) Predicted three-dimensional structure of the GmFRS23 protein. (**D**) Predicted three-dimensional structure of the GmFRS49 protein. Two conserved functional domains are highlighted: the FAR1 DNA-binding domain (yellow) and the MULE transposase domain (red). In the non-conserved regions, the secondary structure elements are colored as follows: blue for α-helices, purple for β-sheets, and orange for loop regions. Corresponding amino acid residue ranges for each domain are indicated.

Furthermore, to gain a more comprehensive understanding of the structural features, three-dimensional models of all 49 GmFRS proteins were predicted and constructed using AlphaFold 3 and were categorized according to the subgroups of the phylogenetic tree (Figure S2). Proteins belonging to the same subgroup displayed similar overall folds. Based on phylogenetic analysis (Figure 1), GmFRS7 and GmFRS12 in soybean were identified as homologs of AtFHY3, while GmFRS23 and GmFRS49 were identified as homologs of AtFAR1, representing key members of the soybean FRS family. Structural elucidation of GmFRS7, GmFRS12, GmFRS23, and GmFRS49 reveals conserved architectural features (Figure 6). It is worth noting that the eight members of subgroup I, which are closely related to Arabidopsis AtFHY3/AtFAR1, all contained a high proportion (close to 50%) of random coils in their structures (Table S3). In contrast, members of subgroup II, closely associated with AtFRS6/AtFRS8, exhibit higher proportions of α-helical structures (>45%). Meanwhile, members of subgroup VI, which are phylogenetically closest to the four Arabidopsis AtFRFs, present a distinctive, continuous α-helical conformation. Based on these findings, we speculate that GmFRS proteins within the same subgroup share similar structural architectures, which may underlie their functional similarities.

### 2.5. Analysis of Cis-Elements in the GmFRS Gene Promoters

**Figure 7:**
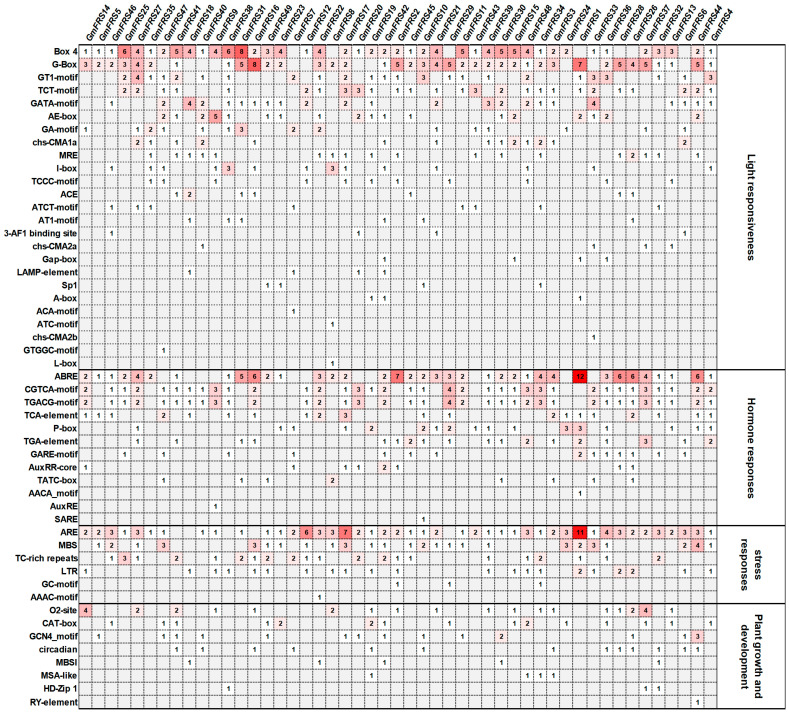
The number of *cis*-elements in the promoter of the *GmFRS* gene family. The color intensity indicates the abundance of each *cis*-element, with darker shades representing higher counts and lighter shades representing lower counts.

To investigate whether the *GmFRS* gene family is transcriptionally regulated in response to various environmental signals, we extracted the putative promoter sequences from the 2000 bp region upstream of each gene and analyzed them using PlantCARE data (Figure 7 and Figure S3, Table S5). A total of 1134 *cis*-regulatory elements were identified, primarily associated with light responses (44.8%), plant hormones (29.4%), stress responses (18.0%), and the growth and development of plants (7.8%). The most abundant elements were light-responsive Box-4. Other frequent elements included ABRE (ABA response) in 37 genes, CGTCA/TGACG motifs (JA response) in 36 genes, ARE (anaerobic induction) in 44 genes, MBS (drought response) in 25 genes, TC-rich repeats (defense) in 21 genes, and LTR (low-temperature response) in 21 genes (Figure 7). Moreover, the presence of *cis*-regulatory elements linked to growth and developmental processes were also detected, suggesting that they might respond to stage-specific signals and regulate expression in particular tissues or developmental stages. These results demonstrate complex *cis*-regulatory networks governing *GmFRS* expression and provide insights into their regulatory mechanisms in light signaling and developmental processes.

### 2.6. Subcellular Localization of Key GmFRS Family Members

**Figure 8:**
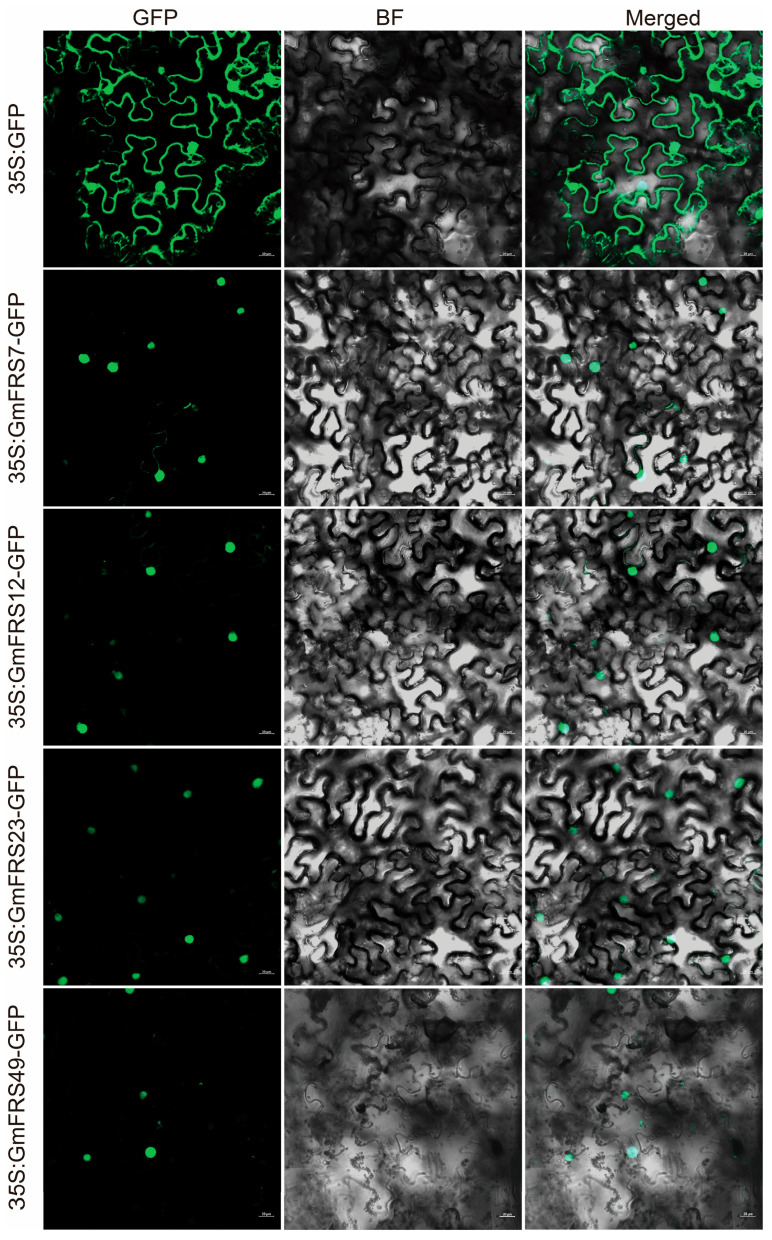
Subcellular localization of GmFRS7, GmFRS12, GmFRS23, and GmFRS49 in *N. benthamiana* leaf epidermal cells. BF, brightfield; GFP, green fluorescent protein. Scale bars, 20 µm.

To gain initial insights into the functions of the soybean FRS family, we generated fusion protein expression constructs for GmFRS7, GmFRS12, GmFRS23, and GmFRS49, transiently expressed them in tobacco (*Nicotiana benthamiana*) leaf cells, and examined their subcellular localization. Fluorescence microscopy revealed that the GmFRS7-GFP, GmFRS12-GFP, GmFRS23-GFP, and GmFRS49-GFP fusion proteins were predominantly localized in the nucleus (Figure 8). This observation is consistent with their predicted subcellular localization and the established localization patterns of their *A. thaliana* homologs AtFHY3/AtFAR1 [16], which supports their predicted role as transcription factors.

### 2.7. Expression Patterns of GmFRS Genes in Different Organs

**Figure 9:**
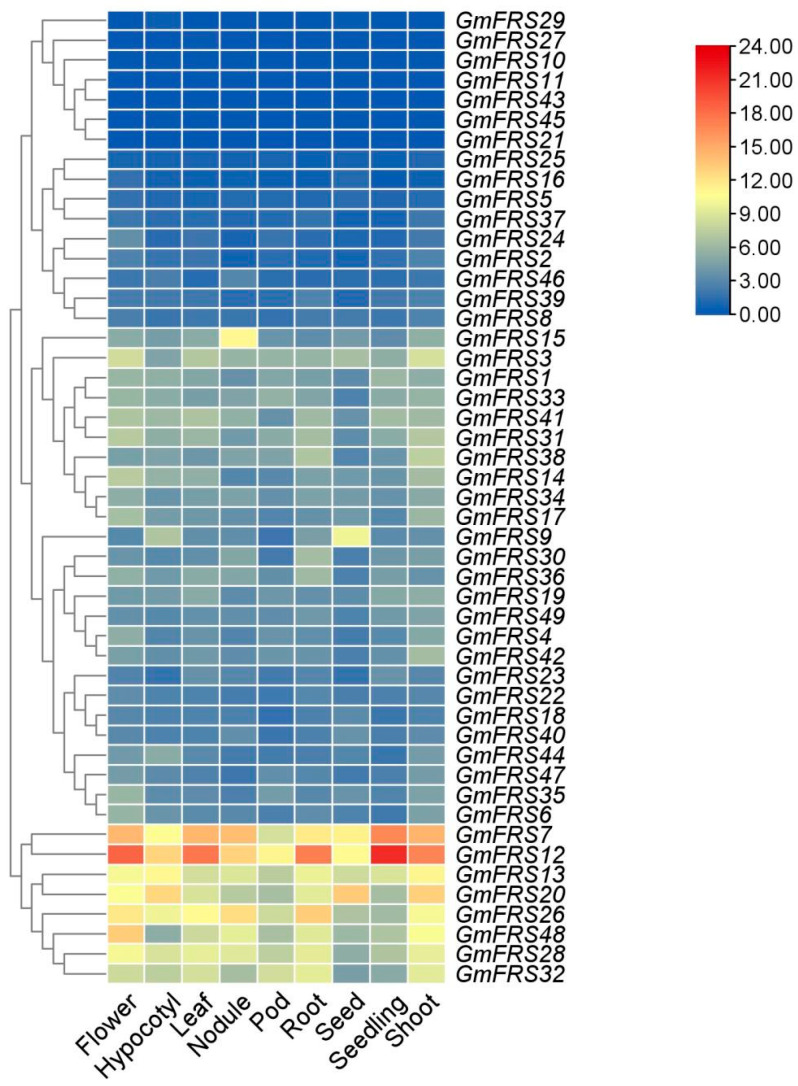
Expression difference in *GmFRS* gene family members in different organs of *Glycine max.* Expression data were retrieved from the publicly accessible Soybean Expression Atlas (SoyX) v2 database.

To investigate the potential function of *GmFRS* genes in soybean growth and development, we analyzed RNA-seq expression profiles across soybean organs, including the flower, hypocotyl, leaf, nodule, pod, root, seed, shoot, and seedling, using Phytozome data (Figure 9, Table S6). The results revealed distinct expression patterns for different *GmFRS* genes in different organs. Specifically, *GmFRS7*, *GmFRS12*, *GmFRS13*, *GmFRS20*, *GmFRS26*, *GmFRS28*, *GmFRS32*, and *GmFRS48* showed relatively high transcript abundance across all examined organs, suggesting potential ubiquitous roles in multiple physiological processes. In contrast, seven genes (*GmFRS10*, *GmFRS11*, *GmFRS27*, *GmFRS29*, *GmFRS43*, and *GmFRS45*) showed a negligible expression. *GmFRS* genes were primarily expressed in the flower, stem, root, and seed (Figure 9, Table S6). Most *GmFRS* genes exhibited preferential expression: 32.7% peaked in flowers, 26.5% in stems, 10.2% in roots, 8.2% in seeds/nodules/seedlings, 4.0% in hypocotyls, and 2.0% in leaves.

### 2.8. Transcriptional Expression of GmFRS Genes During Photoresponse

The presence of light-responsive elements in the promoters indicates that the *GmFRS* family likely plays a key role in light responses. As the red/far-red reflects many environmental cues such as planting density, time of day, and latitude for plants, we explore their response to different red/far-red light ratios by exposing soybean plants to high R:FR (R:FR = 4.5) and low R:FR (R:FR = 0.45) light under long-day conditions (LD). Leaf samples were collected at three time points: 4 h before light treatment (−4 h), at light onset (0 h), and after 4 h of light exposure (4 h). This sampling strategy allowed us to track gene expression changes as the plants transitioned from dark to light conditions and during the initial hours of light exposure. Expression analysis was performed on 42 *GmFRS* genes with detectable transcripts, excluding seven genes (*GmFRS10*, *GmFRS11*, *GmFRS21*, *GmFRS27*, *GmFRS29*, *GmFRS43*, and *GmFRS45*) lacking 5′- and/or 3′-UTRs that showed a negligible expression in organ-specific profiling and failed qPCR amplification.

**Figure 10:**
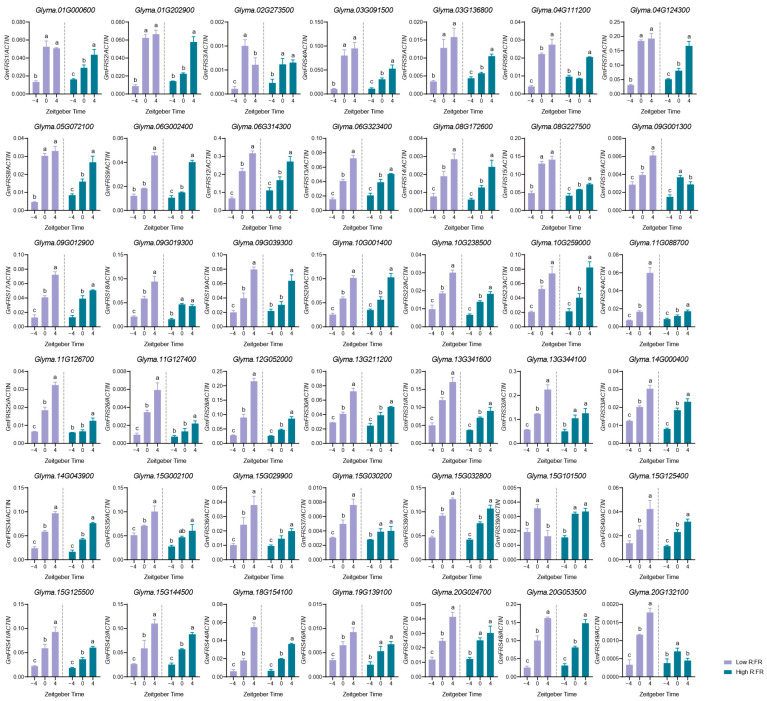
Expression analysis of *GmFRS* genes in soybean leaves under different light (high R:FR; low R:FR). Expression levels of the indicated *GmFRS* genes were determined by quantitative real-time PCR (qPCR). Gene names are indicated on the *Y*-axis of the figure. Different letters above the bars indicate significant differences (*p* < 0.05) as determined by one-way ANOVA (Tukey’s multiple-comparison test). Data are shown relative to the control gene *Actin* and represent means ± SD for three biological replicates.

All the genes responded to far-red light-enriched light. Their expression levels increased when far-red light came. Most of the genes (35/42) were also activated by the continuous presence of far-red light, 5/42 could maintain stable expression under this condition, and only *GmFRS3* and *GmFRS39* exhibited rapid transcriptional induction, followed by partial attenuation after 4 h of continuous irradiation. On the other hand, the red light-enriched conditions could activate almost all the *FRS* genes, except for five exceptions (*GmFRS3*, *GmFRS6*, *GmFRS19*, *GmFRS25*, and *GmFRS26*). Moreover, under continuous red light irradiation, the expression of these genes could also increase or remain stable, except for *GmFRS49*. However, red light’s activation effect on most *FRS* genes was not as pronounced as that of far-red light (except for *GmFRS20*) (Figure 10), which shows that these genes respond to light, especially far-red light. These patterns establish the preferential far-red sensitivity of *GmFRS* genes and their functional association with shade avoidance regulation.

Within the soybean *FRS* gene family, *GmFRS12*, *GmFRS23*, *GmFRS34*, *GmFRS38*, and *GmFRS48* exhibited similar responsiveness to both red and far-red light, showing activation under both conditions, which indicates their potential involvement in general light-signaling pathways. In contrast, *GmFRS7*, *GmFRS15*, *GmFRS24*, *GmFRS28*, *GmFRS36*, and *GmFRS49* displayed preferential sensitivity to far-red light compared to their response under red light, suggesting that these six genes may play primary roles in shade avoidance responses.

## 3. Discussion

The FRS family comprises transcription factors derived from MULE transposases, which have been domesticated over the evolutionary to play key roles in plant light signaling, growth regulation, stress responses, and other developmental processes [24]. In Arabidopsis thaliana, 18 FRS members have been identified and functionally characterized. Subsequent studies have reported the presence of the FRS family in diverse plant species, including maize, eucalyptus, potatoes, cucumbers, and tea. In this study, we systematically identified 49 *GmFRS* family members in soybean (*Glycine max*) and analyzed their phylogenetic relationships, gene structures, physicochemical properties, *cis*-regulatory elements, and organ-specific expression patterns, as well as their transcriptional responses under different light conditions.

### 3.1. Phylogenetic Analysis of the GmFRS Genes

In this study, we identified 49 *GmFRS* genes randomly distributed across all 20 soybean chromosomes, which phylogenetic analysis classified into seven subgroups (Figure 1, Table S1). Recent work first implicated soybean FRS homologs in cold-stress responses, and reported 72 *FRS* candidate genes in soybean [29]. When we compared this list with our list, 25 of the reported loci could not be retrieved, while we additionally identified two previously unreported *FRS* genes (*Glyma.12G051250* and *Glyma.13G000732*) that carry the conserved FRS domain and are present in the updated genome assemblies (Table S1). Further analysis revealed that 25 of these genes were only present in the older *Glycine max* Wm82.a2.v2 genome assembly but were absent in the updated *Glycine max* Wm82.a2.v4 and *Glycine max* Wm82.a2.v6 assemblies and exhibited negligible expression across examined tissues [29]. These observations suggest that the 25 loci are pseudogenes—most likely artifacts stemming from early assembly errors or annotation inaccuracies.

Synteny analysis revealed the presence of segmental duplications within the *GmFRS* family, which may have contributed to its evolution and functional diversification. Furthermore, Ka/Ks analysis indicated that these genes have undergone purifying selection during evolution, a pattern consistent with observations in grapes and potatoes [27,28]. Compared with the 18 *FRS* genes in *A. thaliana*, soybean carries 49, a product of successive polyploidies—ancient Gamma WGT, Legume WGD, and Glycine WGD or segmental duplications [39]—which yielded only 15 syntenic pairs (29 genes). The soybean FRS family exhibits remarkably low synteny conservation, significantly below typical expansion levels for legume gene families. This reduced synteny phenomenon recurs in monocot species such as barley (*Hordeum vulgare*) [40], indicating cross-species evolutionary conservation. Critically, the transposase-derived domains in FRS proteins have undergone functional decay during domestication [41]. Consequently, extant non-syntenic genes likely represent degraded remnants of ancient duplication events rather than products of recent transposable element-mediated replication. Inter-species collinearity analysis revealed that soybean *FRS* genes exhibit syntenic blocks in eudicots but not in monocots, suggesting that the *FRS* gene family likely originated after the divergence of monocots and eudicots (Figure 3B; Table S9).

Phylogenetic analysis revealed that members of subgroup VII, which lacks an Arabidopsis representative, form a distinct clade with FRS proteins from tomato and maize, suggesting a potential functional conservation and mechanistic similarity among these orthologs. The consistent absence of Arabidopsis members in this subgroup is also observed in the grape FRS family [27]. AtFHY3 and AtFAR1 share the closest affinity with GmFRS7/12 and GmFRS23/49, respectively (bootstrap > 85%). These GmFRS members are tentatively inferred as putative orthologs, potentially retaining conserved functions in soybean (Figure 1). However, future work should include expression profiling, complementation assays in *A. thaliana* mutants, or CRISPR knockout in soybean to confirm orthology and functional conservation.

### 3.2. The Physicochemical Properties and Structure of the GmFRSs

Physicochemical property analysis revealed that most GmFRS proteins exhibited instability, consistent with previous findings in Arabidopsis [9], except for GmFRS2 and GmFRS36. Subcellular localization predictions indicated that nearly all GmFRS members were localized to the nucleus (Table S2).

The identified GmFRS family members in soybean exhibit variations in conserved domains, consistent with findings in Arabidopsis [42]. For instance, GmFRS25, GmFRS45, and GmFRS21 lack the SWIM zinc finger domain, while GmFRS10 retains only the MULE transposase domain. Proteins in subgroup VI are all truncated forms, similar to those in Arabidopsis, containing only the FAR1 DNA-binding domain composed of motif 2 and motif 7 (Figure 4A) and lacking both the MULE transposase domain and the SWIM zinc finger domain (Figure 4B). In Arabidopsis, full-length FRS proteins such as FHY3/FAR1 typically function as transcriptional activators, whereas truncated FRF proteins may negatively regulate their activity by competing for DNA-binding sites [42]. Notably, FRS7 and FRS12 contain two DNA-binding domains and regulate the flowering time by binding to the promoters of downstream genes *GIGANTEA* and *PIF4* [43]. Thus, these domain differences suggest potential functional diversification among GmFRS proteins, providing a structural basis to act as a central hub integrating light signaling, hormone signaling, developmental programs, and stress responses, thereby enabling the precise regulation of plant life processes.

Variations in gene structures serve as an important indicator for assessing the evolutionary relationships and functional diversification of gene families. We observed a clear correlation between exon–intron architecture and the composition of conserved protein domains. Gene structure analysis revealed that genes within the same subgroup share similar exon–intron organizations. Notably, most members are characterized by a single long exon. However, four members in subgroup V—*GmFRS17*, *GmFRS20*, *GmFRS19*, and *GmFRS42*—exhibit a concise structure composed of three exons, with each gene containing three complete conserved domains. This finding suggests a modular encoding principle of “one exon, one independent domain” and implies that exon shuffling may have been a significant evolutionary mechanism for this family [44,45]. In some members of clades IV and VI, we identified remarkably elongated intron sequences. These hyper-long intronic regions are likely enriched with complex *cis*-regulatory elements (such as enhancers, silencers, or non-coding RNA genes), potentially enabling genes in these subgroups to integrate more diverse and precise environmental signals (e.g., biotic/abiotic stresses) or developmental cues, thereby conferring a more complex transcriptional regulatory potential [46].

Moreover, the gene structure is instrumental in transcriptional regulation and in functional diversification [47,48]. Analyses of tissue expression patterns through RNA-seq indicated seven *GmFRS* members (*GmFRS10*, *GmFRS11*, *GmFRS21*, *GmFRS27*, *GmFRS29*, *GmFRS43*, and *GmFRS45*) that exhibited nearly undetectable expression levels (Figure 9, Table S6) and were annotated as lacking both 5′ and 3′ UTRs. We speculate that the absence of UTRs may have led to the loss of regulatory elements and structures essential for mRNA stability and translation [49], thus potentially driving the evolution of these genes into pseudogenes.

Secondary structure analysis revealed that GmFRS proteins are primarily composed of α-helices and random coils (Table S3). Previous studies in Arabidopsis have confirmed the homodimerization of FHY3 and FAR1 [6]. The high proportion of helical and coil structures in soybean FRS proteins suggests that they may similarly form homodimers or heterodimers and interact with various partner proteins to regulate signal transduction. Furthermore, three-dimensional structure predictions of GmFRS proteins support the phylogenetic grouping. The results show that members within the same subgroup share similar folding conformations, indicating a conserved molecular architecture, consistent with observations in the FRS family of grape [27].

### 3.3. The Promoters of GmFRSs Contain Abundant Light and Stress Response Elements

Analysis of the *cis*-regulatory elements in the *GmFRS* promoter regions revealed an abundance of elements related to light response, abiotic stress response, and hormone response (Figure 4). Notably, among the light-responsive elements, the Box-4 *cis*-element was particularly prevalent, consistent with findings in potato [28], followed by the G-Box, a key regulatory hub in plants for integrating diverse signals such as light, hormones, and stress. The ARE (anaerobic response element) was present in the promoters of nearly all genes, suggesting that the FRS family may be involved in abiotic stress processes induced by salt, low temperature, and anaerobiosis. Furthermore, hormone-responsive elements, including ABRE and the CGTCA/TGACG motifs, were widely distributed in the promoters, indicating potential roles for the soybean FRS family in abscisic acid (ABA) and jasmonic acid (JA) signaling pathways, which are often crucial for plant responses to abiotic stress [50]. The complex combination of *cis*-elements in the *GmFRS* promoter regions enables these genes to integrate and respond to diverse signals, including light, ABA, JA, drought, cold, and defense cues [51].

### 3.4. Expression Patterns of GmFRS Genes in Different Organs and Their Responses to Different Light Conditions

Investigating the expression patterns of *FRS* family genes in various soybean organs is crucial for deciphering their functions. Expression analysis revealed distinct organ-specific patterns among *GmFRS* genes, suggesting potential stage- and tissue-specific roles. Overall, *GmFRS* transcripts were most abundant in flowers (32.7%), followed by stems (26.5%). Notably, the expression level of *GmFRS12* was significantly higher than that of all other members across all of the organs examined. Furthermore, *GmFRS13*, *GmFRS26*, *GmFRS32*, and *GmFRS28*, all belonging to subgroup VI, exhibited a relatively high transcript abundance in various organs. Although proteins in this subgroup are truncated forms, their promoter regions contain *cis*-regulatory elements distributed across four functional categories: light response, hormone response, stress response, and plant growth/development. This suggests that these genes may be extensively involved in and play important roles in diverse physiological processes during soybean growth. Functional studies of this subgroup are likely a key direction for understanding the *GmFRS* family in soybean. Additionally, we examined the responses of *GmFRS* genes to light conditions with varying ratios of red (R) to far-red (FR) light. All genes responded to both red and far-red light irradiation, which is consistent with the presence of light-responsive *cis*-acting elements enriched in their promoter regions. However, most genes showed a stronger sensitivity to FR light, particularly *GmFRS7*, *GmFRS15*, *GmFRS24*, *GmFRS28*, *GmFRS36*, and Gm*FRS49*. These six genes may play a leading role in shade avoidance responses [21,52,53].

## 4. Materials and Methods

### 4.1. Plant Material and Growth Conditions

Soybean plants (Williams 82) were grown in a controlled-environment chamber at 25 °C under a 16 h light/8 h dark photoperiod. Soybean seeds were germinated in vermiculite for 5 days and then transferred to conditions of either high R:FR (150–180 μmol·m^−2^·s^−1^ red light/30–40 μmol·m^−2^·s^−1^ far-red light) or low R:FR (150–180 μmol·m^−2^·s^−1^·red light/280–320 μmol·m^−2^·s^−1^ far-red light) for an additional 20 days. To capture the changes in *GmFRS* level of expression at the transition from dark to light, leaf samples were collected 4 h before light onset (−4 h), at the onset of light treatment (0 h), and 4 h after light onset (4 h). Samples were immediately wrapped in aluminum foil, flash frozen in liquid nitrogen, and stored at −80 °C. Total RNA was extracted from the leaf samples, with three biological replicates prepared per sample.

### 4.2. Identification of GmFRS Gene Family Members

To identify FRS homologs in soybean, we employed a combination of homology search and structural domain validation. Genomic data, annotation files, and protein sequences for soybean (*Glycine max Wm82.a2.v2*, *Glycine max Wm82.a2.v4*, and *Glycine max Wm82.a2.v6*) were obtained from the Phytozome 13 database. Using the BLAST algorithm within the online tool TBtools v2.322 [32], the soybean protein database was scanned using *A. thaliana* FHY3/FAR1 family proteins as the query template. High-quality candidate GmFRS proteins were selected based on a sequence identity > 50% and an E-value < 1.0 × 10^−5^. The hidden Markov model (HMM) profile for the FHY3/FAR1 (FRS) family was downloaded from the Pfam database (http://pfam.xfam.org/, accessed on 20 November 2024). The resulting sequences were subsequently validated for functional domains using the NCBI Conserved Domain Database (CDD; https://www.ncbi.nlm.nih.gov/cdd/, accessed on 21 November 2024) and the InterPro website (https://www.ebi.ac.uk/interpro/, accessed on 21 November 2024), leading to the identification of 49 candidate genes. All gene names are listed in Table S1.

### 4.3. Prediction of Physicochemical Properties of GmFRS Proteins

Physicochemical properties of the GmFRS protein, including polypeptide chain length, relative molecular mass, theoretical isoelectric point (pI), and hydrophobicity index (GRAVY), were analyzed using the ProtParam tool on the ExPASy website (https://web.expasy.org/protparam/, accessed on 21 December 2024). Subsequently, the subcellular localization propensity of GmFRS was predicted using the online tool DeepLoc-2.1 (https://services.healthtech.dtu.dk/services/DeepLoc-2.1/, accessed on 22 January 2026) and CELLO (https://cello.life.nctu.edu.tw/, accessed on 22 January 2026). All resulting data were integrated and are summarized in Table S2.

### 4.4. Phylogenetic Analysis of GmFRS Gene Family Members

FHY3/FAR1 protein sequences from *A. thaliana* (TAIR 10), *Z. mays* (RefGen_V4), and *S. lycopersicum* (ITAG2.4) were retrieved from the plant genome databases TAIR (http://www.arabidopsis.org/, accessed on 23 November 2024) and Phytozome (http://www.phytozome.net/, accessed on 20 November 2024). All gene names are listed in Table S8. Multiple sequence alignment of the FHY3/FAR1 family protein sequences from *A. thaliana*, *S. lycopersicum*, *Z. mays*, and *G. max* was performed using MEGA v.11 [54]. Subsequently, a phylogenetic tree was constructed using the Maximum Likelihood (ML) method in MEGA v.11; bootstrap analysis with 1000 replicates was applied to assess the reliability of the tree topology. Finally, the phylogenetic tree was visually refined using the online iTOL tool v7 (https://itol.embl.de/, accessed on 24 November 2024).

### 4.5. Visualization of Gene Structure, Domain, and Conserved Motif of GmFRS Family Members

Using soybean Genome Feature Format (GFF) files, we constructed a gene structure map for the 49 *GmFRS* family members using the TBtools software locally. After extracting the protein sequences of *GmFRS* genes, conserved motifs were predicted and analyzed using the online MEME tool v5.5.6 [55], with the maximum motif count set to 10, to obtain motif information; the MEME results were visualized using TBtools. Gene structure analysis using TBtools predicted the positions and numbers of exons and introns for each *GmFRS* gene. Conserved domain information for the *GmFRS* protein family members was visualized using TBtools v2.322. Protein sequences were submitted to the NPS@ online tool (https://npsa.lyon.inserm.fr/cgi-bin/npsa_automat.pl?page=/NPSA/npsa_sopma.html, accessed on 22 January 2026) for secondary structure prediction. Multiple sequence alignment for protein was performed using DNAMAN. The three-dimensional structures were predicted by AlphaFold3 and visualized using PyMOL v3.1.6.1.

### 4.6. Analysis of Cis-Acting Elements of GmFRS Gene Family Members

The genomic sequences of the *GmFRS* genes were based on the soybean reference genome (*Glycine max Wm82.a4.v1*) from the Phytozome 13 database. For promoter analysis, we extracted the 2000 bp genomic sequences upstream of the predicted transcription start site (TSS) for each gene. The TSS was defined as the 5′-most nucleotide of the primary transcript model for each *GmFRS* gene in the Phytozome GFF3 annotation file. Putative *cis*-regulatory elements within these promoter regions of all 49 *GmFRS* genes were identified using the PlantCARE online database (http://bioinformatics.psb.ugent.be/webtools/plantcare/html/, accessed on 21 November 2024). Visualization and comprehensive analysis of the identified *cis*-regulatory elements—including their category, distribution, and frequency—were performed using TBtools.

### 4.7. Chromosomal Localization and Synteny Analysis of GmFRS Gene Family Members

The physical chromosomal positions of all 49 *GmFRS* genes were obtained from Phytozome. The soybean genome and annotation files were imported into TBtools, and gene location mapping was performed using the software’s Chromosome Distribution Module. The collinearity relationship between different gene pairs was performed using the ‘One step MCScanX’ function in TBtools (E-value < 1 × 10^−10^, Num of BlastHits = 5). The results were visualized using the Dual Systeny Plot for MCScanX package in the TBtools. The Ka/Ks of all tandemly duplicated gene pairs were calculated using KaKs_Calculator 2.0 [56].

### 4.8. Differences in the Expression of GmFRS Gene Family Members

Expression data for the *GmFRS* gene family across multiple organs (flower, hypocotyl, leaf, nodule, pod, root, seed, seedling, and shoot) was downloaded from the Soybean Expression Atlas v2 online platform (https://soyatlas.venanciogroup.uenf.br, accessed on 5 March 2025). Subsequently, a heatmap illustrating the expression patterns of *GmFRS* genes in these organs/tissues was constructed using TBtools.

### 4.9. RNA Isolation and Expression Analysis of GmFRS Genes of Light Response

Total RNA was extracted from soybean leaves using an RNA extraction kit (Cowin Biotech, Taizhou, China) following the manufacturer’s protocol. cDNA was synthesized with a reverse transcription kit (GenStar, Beijing, China). Gene expression patterns under high/low R:FR conditions were analyzed by quantitative real-time PCR (qPCR) using a detection system (Analytik Jena GmbH, Jena, Germany). Primer sequences are provided in Table S7.

### 4.10. Subcellular Localization Analysis

The coding sequences of *GmFRS7*, *GmFRS12*, *GmFRS23*, and *GmFRS49* were amplified and in-frame fused to the C-terminus of GFP in the pTF101 vector, generating the constructs 35S:GFP-GmFRS7, 35S:GFP-GmFRS12, 35S:GFP-GmFRS23, and 35S:GFP-GmFRS49. The resulting constructs were introduced into *Agrobacterium tumefaciens* strain GV3101 and then infiltrated into *Nicotiana benthamiana* leaves for transient expression. After 2 days, infiltrated leaf epidermal cells were imaged using a confocal laser scanning microscope (LSM800; Zeiss, Oberkochen, Germany).

## 5. Conclusions

This study presents a genome-wide analysis of the *FRS* gene family in soybean, leading to the identification of 49 *GmFRS* genes. Comprehensive analyses were conducted on their physicochemical properties, phylogenetic relationships, gene structures, conserved motifs, conserved domains, gene duplication events, collinearities, promoter *cis*-regulatory elements, and expression profiles. The *GmFRS* genes were classified into seven subfamilies, with members within each subfamily sharing conserved motif compositions and similar exon/intron structures. Protein tertiary structure predictions further revealed conserved three-dimensional architectures within subfamilies. Promoter analysis indicated that cis-regulatory elements are predominantly associated with light response, abiotic stress, and hormone response. Expression profiling demonstrated that *GmFRS* genes are differentially expressed across various soybean tissues, with notably high transcript levels in flowers, and most members exhibit a strong response to far-red light. These findings establish a foundation for further exploration of *GmFRS* gene functions and their roles in light-signaling regulatory networks, providing theoretical insights for the improvement of agronomic traits in soybean.

Based on the findings of this study, future studies should aim to validate the functions of core candidate genes (e.g., *GmFRS7*, *GmFRS12*, *GmFRS23*, and *GmFRS49*) through in vivo CRISPR/Cas9 knockout, coupled with *in vitro* analyses of their DNA-binding specificity and protein–protein interactions.

## Data Availability

All data generated or analyzed in this study are included in the main text and its Supplementary Materials.

## Supplementary Materials

The following supporting information can be downloaded at: https://www.mdpi.com/article/10.3390/ijms27062638/s1.
