# Supplementary material for: Genome-Wide Analysis of FAR-RELATED SEQUENCES (FRS) Genes Related to Light Response in Soybean (Glycine max)
Source: Int J Mol Sci. 2026 Mar 13;27(6):2638. doi: 10.3390/ijms27062638 (PMC13026280; doi:10.3390/ijms27062638)
Supplement: Supplementary file 1 [file ijms-27-02638-s001.zip › Supplement Figures.pdf]

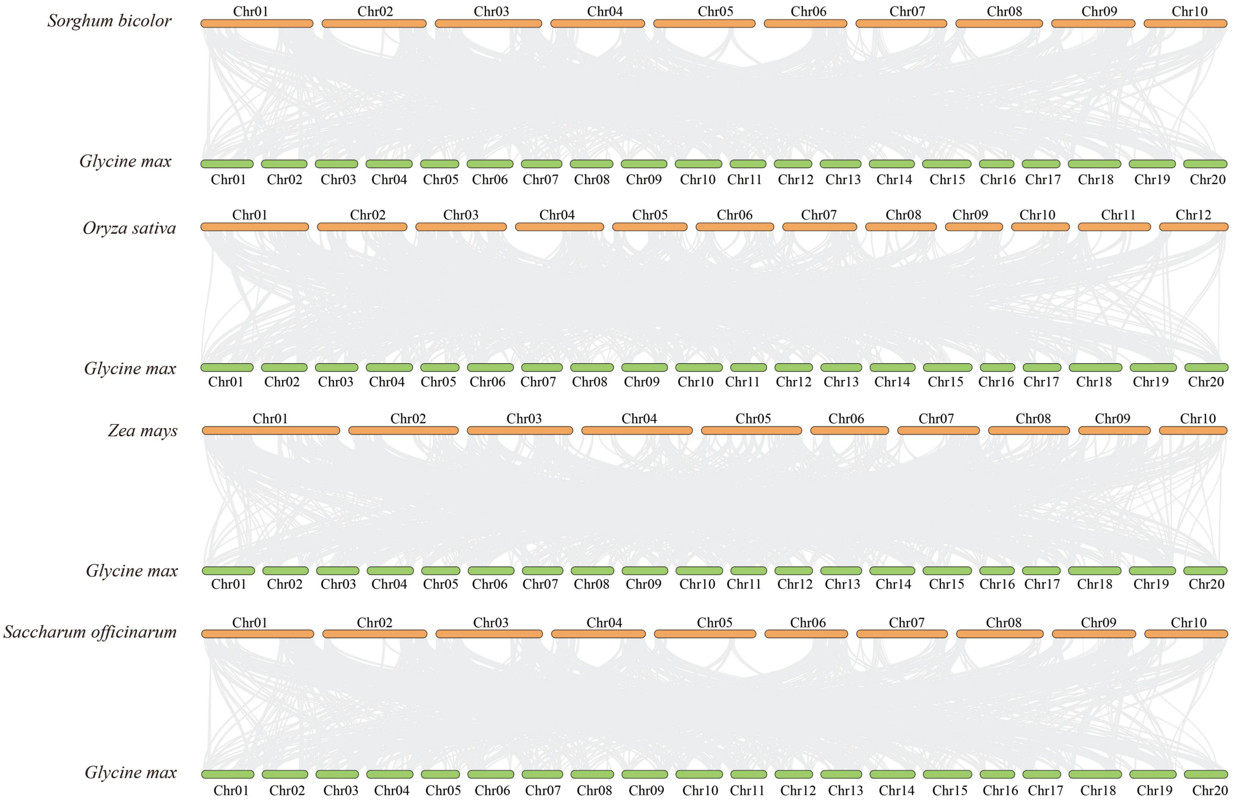

Figure S1. The collinearity analysis of the *GmFRS* family members between soybean and four monocots, including *Sorghum bicolor, Oryza sativa, Zea mays, Saccharum spontaneum.* The gray lines in the background depict all homologous gene pairs identified by BLASTP (E-value < 1e-10, Num of BlastHits = 5). The absence of any highlighted lines (in red) connecting *GmFRS* gene loci to monocot chromosomes indicates that no collinear blocks containing *GmFRS* genes were detected.

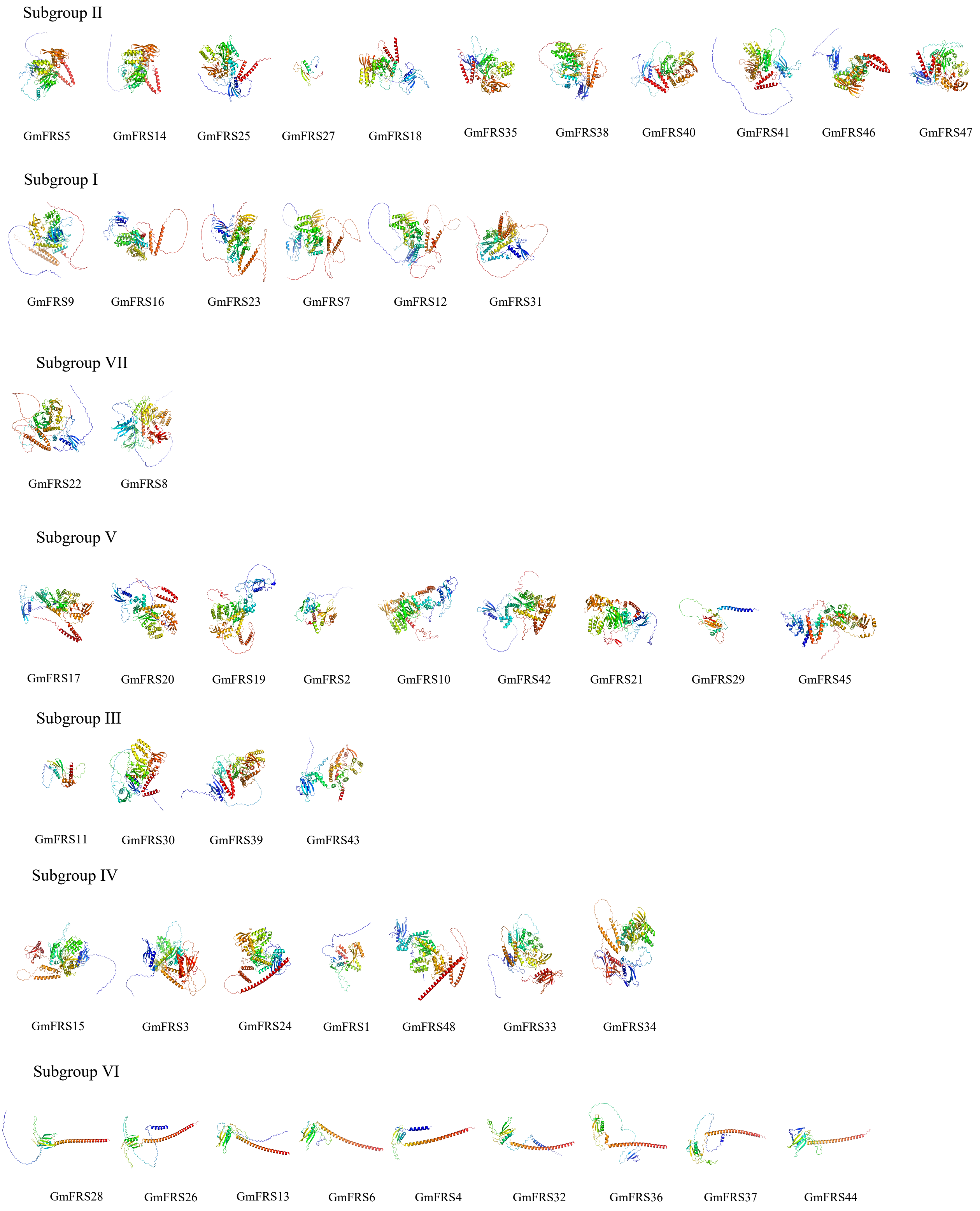

Figure S2. Three-dimensional models of GmFRS proteins. The color gradient from blue to red in the figure represented the sequential arrangement of protein structures from N-terminal to C-terminal.

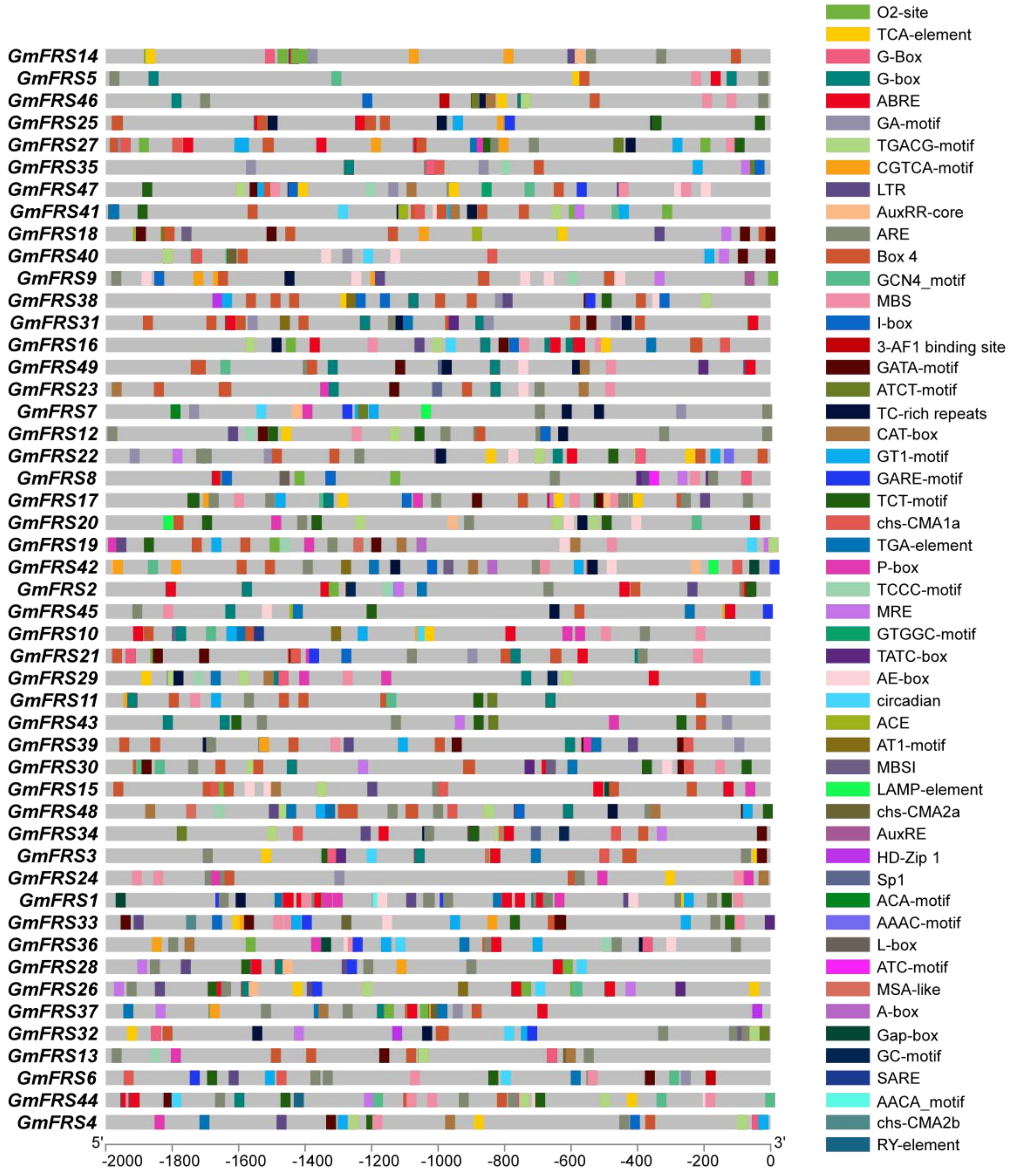

Figure S3. Schematic of cis-element in the promoter of *GmFRS*s.
